# Retinal Neurodegeneration: Correlation between Nutraceutical Treatment and Animal Model

**DOI:** 10.3390/nu13030770

**Published:** 2021-02-27

**Authors:** Ilaria Piano, Mattia Di Paolo, Francesca Corsi, Eugenia Piragine, Silvia Bisti, Claudia Gargini, Stefano Di Marco

**Affiliations:** 1 Department of Pharmacy, University of Pisa, Via Bonanno, 6, 56121 Pisa, Italy; ilaria.piano@unipi.it (I.P.); mattia.dipaolo@univaq.it (M.D.P.); francesca.corsi@in.cnr.it (F.C.); eugenia.piragine@farm.unipi.it (E.P.); gargini@farm.unipi.it (C.G.); 2 Interuniversity Consortium -Biostructures and Biosystems National Institute (INBB), Via Medaglie d’Oro 305, 00136 Roma, Italy; s.bisti@team.it; 3 Institute of Neuroscience—CNR, Via Giuseppe Moruzzi 1, 56124 Pisa, Italy; 4 Center for Synaptic Neuroscience and Technology, Istituto Italiano di Tecnologia (IIT), 16132 Genova, Italy; 5 Interdepartmental Research Center “Nutraceuticals and Food for Health”, University of Pisa, 56121 Pisa, Italy

**Keywords:** retinal degeneration, oxidative stress, nutraceutical compounds, preventative therapy

## Abstract

Retinal diseases can be induced by a variety of factors, including gene mutations, environmental stresses and dysmetabolic processes. The result is a progressive deterioration of visual function, which sometimes leads to blindness. Many treatments are under investigation, though results are still mostly unsatisfactory and restricted to specific pathologies, particularly in the case of gene therapy. The majority of treatments have been tested in animal models, but very few have progressed to human clinical trials. A relevant approach is to study the relation between the type of treatments and the degenerative characteristics of the animal model to better understand the effectiveness of each therapy. Here we compare the results obtained from different animal models treated with natural compounds (saffron and naringenin) to anticipate the potentiality of a single treatment in different pathologies.

## 1. Introduction

Nowadays, poor vision and blindness affect more than 39 million people; this number is continuously growing with an increasing world population [1,2]. Vision loss can be triggered by diseases affecting different regions of the eye, but most of these disorders are concentrated in the retina and can lead to irreversible blindness. Retinal neurodegenerations are a group of heterogeneous disorders that have different etiology but present similar pathological features that are associated with the uniqueness of retinal tissue. In particular, the hyper-specialization of the retina requires a high metabolic activity [3] which is finely assisted by the strict immunosurveillance [4], the resilience pathways [5,6,7] and the supportive cells (Retinal Pigment Epithelium, RPE; Müller cells) [8,9,10]. This delicate physiological homeostasis might easily be disrupted, leading to burdensome pathological features whose dynamic interactions describe a specific profile, depending on the etiopathogenesis of the disease.

To cope with degenerative processes, an approach that has raised considerable interest is to target oxidative stress, which has been linked to several pathological events [11]. Its related sequence of developments includes the production of reactive oxygen species (ROS) that react with biological molecules, causing changes in normal biological functions and leading to neuroinflammation, degeneration and cell death. The retina is a tissue particularly vulnerable to changes in oxygen concentration and, consequently, to oxidative stress because it is rich in polyunsaturated lipid membranes and has a high aerobic metabolism. In different retinal diseases, oxidative stress might play distinct roles: In age-related macular degeneration (AMD), classified as a multifactorial disease, oxidative stress may be one of the leading causes of dysmetabolic events, the production of misfolded proteins and drusen deposition. In other neurodegenerative diseases, such as Retinitis Pigmentosa (RP), the first event is due to a genetic mutation while oxidative stress becomes relevant in driving the progression of the disease. Specifically, in RP, following the death of rods, hyperoxia increases in the outer retina giving rise to the secondary phase of the pathology, characterized by cone degeneration. In the rd10 animal model, as discussed in the literature [12,13], one of the hypotheses for the secondary death of cones is the reduction in oxygen consumption due to the loss of rods. The decrease in oxygen utilization causes an increase in ROS production which renders the tissue environment unfavorable for cell survival (increased oxidation of macromolecules such as nucleic acids, proteins and lipids, and the reduction of antioxidant defense systems), with consequent degeneration and death of cones [14,15,16,17].

Accordingly, despite the variety of factors that induce neurodegeneration, shared pathways such as oxidative stress might accelerate its progression. On this basis, the AREDS protocol, an antioxidant formulation tested in AMD, was developed (see for ref. [18]). Recently, much interest has gathered around the neuroprotective efficacy of natural molecules, which can be singularly extracted or used crude. Naringenin and quercetin are examples of natural and isolated molecules that might be effective in treating diabetic retinopathy [19,20]. The efficacy of chronic antioxidant treatment with naringenin and quercetin [21,22]) to preserve cone morphology and functionality was recently assessed in the RP model, rd10 mice 17. In this context, treatment with nutraceutical molecules fails to save the rods that are genetically programmed to die.

On the other hand, ratios among molecules in the entire natural compound might represent an added value for neuroprotection. A natural compound, such as saffron, shows unexpected properties that exceed its general use as an antioxidant. This spice, probably through an integrated and multilevel mechanism, can preserve visual function in animal models of retinal diseases [23,24,25], as well as in patients with AMD [26,27,28,29] and ABCA4-Related Stargardt Macular Dystrophy [30,31]. The complexity of this coordinate mechanism nonetheless requires a harmonic association between treatments and the proper pathological profile. The efficacy of a neuroprotective treatment in a particular retinal disease might not be of the same entity in even a slightly different context.

From all these studies, the question arises whether it is achievable to define the best possible correlation between the type of treatment and the specific pathological profile where treatments might fully express their neuroprotective potential. Here, we focus on two promising natural products already tested for their neuroprotective capacity; saffron and naringenin.

We aim to underline “key” correlations between the type of treatment and the degenerative characteristics of the animal model used to reveal the potential efficacy of a single treatment in selected retinal pathologies.

## 2. Materials and Methods

### 2.1. Animals

C57BL/6J male mice were treated according to Italian and European institutional guidelines, following experimental protocols approved by the Animal Welfare Organization (OPBA) of the University of Pisa and the Italian Ministry of Health (Protocol #653/2017-PR-DB173.3.EXT.0, Department of Pharmacy, University of Pisa). Mice were regularly fed ad libitum with a standard diet (4RF18, Mucedola srl, Milan, Italy) and bred at a lighting level below 60 lux with dark-light cycles of 12:12 h. All experiments were conducted under deep anesthesia by intraperitoneal injection (i.p.) of 20% urethane in a saline buffer (0.9% NaCl) at a dose of 0.1 mL/10 g body weight. At the end of the experimental procedure, the animals were sacrificed by cervical dislocation, maintaining the condition of deep anesthesia.

Royal College of Surgeons (RCS) and RCS-rdy rats: all procedures were in conformity with the ARVO Statement for the Use of Animals in Ophthalmic Research and authorized by the Ministry of Health (authorization number 83/96-A of 29/11/1996). Rats were regularly fed ad libitum with a standard diet (4RF18, Mucedola srl, Milan, Italy) and bred at 5 lux with dark-light cycles of 12:12 h.

Fischer rats: all experiments were conducted following the ARVO Statement for the Use of Animals in Ophthalmic Research and authorized by the Ministry of Health (authorization number 862/2018-PR). Rats were regularly fed ad libitum with a standard diet (4RF18, Mucedola srl, Milan, Italy) and bred at 5 lux with dark-light cycles of 12:12 h.

All rats were humanely sacrificed with carbon dioxide and the eyes[M1] were explanted.

### 2.2. Nutraceutical Treatments

#### 2.2.1. Naringenin

Treatment with naringenin was performed by oral administration in the drinking water. Stock solutions of naringenin (40 mg/mL in DMSO) were prepared daily in order to avoid oxidation phenomena. Naringenin was dissolved in water up to 0.4 mg/mL to obtain the daily dose of 100 mg/kg [17,32]. The dose of Naringenin used in the present study is far from the toxicity values of the molecule which has an estimated LD50 > 5000 mg/kg [33] furthermore, water intake was daily monitored and mice were weakly weighted.

6-month-old C57BL/6J male mice were randomly assigned to four groups: (1) and (2) treated respectively with naringenin (100 mg/kg/day) or vehicle alone (control group) up to 9 months of age (*n* = 6 for each group); (3) and (4) treated respectively with naringenin (100 mg/kg/day) or vehicle alone (control group) up to 12 months of age (*n* = 6 for each group).

#### 2.2.2. Saffron

Animals were daily treated with an aqueous extract of saffron (Saffron REPRON, patent: W02015/145316) (1 mg/kg/day) as a diet supplementation. In RCS rats the treatment started from their prenatal period (at least 1 week before birth), by feeding pregnant animals with the aqueous extract. Saffron dosage as a treatment is completely safe, as demonstrated in previous pharmacological studies [34].

RCS rats were assigned to three groups: (1) RCS rats as control (*n* = 45); (2) RCS rats + Saffron treatment (*n* = 45); (3) RCS-rdy as congenic control (*n* = 20). Each group was analyzed at different time points (P20, P24, P27, P30, P35) and humanely sacrificed.

Fischer rats were assigned to four groups: (1) light damaged (LD) rats, LD control group (*n* = 6) were exposed to 1000 lux for 18 h and left to recover for 15 days; (2) Saffron treatment of light-exposed rats (*n* = 6) started 7 days before LD and continued uninterruptedly until 15 days of recovery; (3) Saffron treatment of Fischer rats (*n* = 3) for 15 days; (4) Untreated control group (*n* = 5).

### 2.3. Electroretinogram (ERG) Recordings

#### 2.3.1. Mice

The general procedures for animal preparation, anesthesia, ERG recording, light stimulation, and data analysis have been described previously in detail [35]. Animals were first dark-adapted overnight and then anesthetized via intraperitoneal injection of Urethane 20% at a dose of 0.1 mL/10 g body weight; ERGs were recorded in complete darkness using coiled gold electrodes contacting the cornea moisturized by a thin layer of gel (Lacrinom, Farmigea, Pisa, Italy), and the reference electrode was inserted at scalp level. Responses were amplified differentially, bandpass filtered at 0.1–500 Hz, digitized at 12.8 kHz by a computer interface (LabVIEW 6.1; National Instruments, Austin, TX, USA) and stored on a disk for processing.

The animal was placed inside a Ganzfeld sphere, 30 cm in diameter, whose interior surface was coated with a highly reflective white paint and exposed to light stimulation. The light stimulation was carried out with a white light electric flash (SUNPACK B3600 DX, Tecad Company, Tokyo, Japan) and six calibrated neutral density filters were used to modulate the intensity.

For scotopic ERG recordings, mice were presented with a single flash of increasing intensity (0.0041 to 83.7 cd×s/m^2^, 0.6 log unit steps), each repeated six times, with an inter-stimulus interval ranging from 20 s for dim flashes to 45 s for the brightest flashes. Isolated cone (photopic) components were obtained by superimposing the test flashes (0.016 to 377 cd×s/m^2^), on a steady background of saturating intensity for rods (30 cd/m^2^) after at least 15 min from background onset. The amplitude of the a-wave was measured at 7 ms after the onset of the light stimulus, and the b-wave was measured from the peak of the a-wave to the peak of the b-wave. Data were analyzed with the LabVIEW 2019 program (National Instruments, Austin, TX, USA).

To estimate the required sample size, we used a conservative approach by paired Wilcoxon-Mann-Whitney test (group of treated animals vs. control animals), considering the amplitude of the ERG waves as the effect size. The required sample size was 6 animals per group. To evaluate the normal distribution of the data obtained, always in terms of ERG waves amplitude, we used the Shapiro-Wilk test.

#### 2.3.2. Rats

The general procedures for animal preparation, anesthesia, ERG recording, light stimulation, and data analysis have been described previously in detail [36]. Animals were first dark-adapted overnight and then anesthetized via intraperitoneal injection of mixed 100 mg/kg ketamine and 12 mg/kg Xylazine (Ketavet 100 mg/mL, Intervet production srl; Xylazine 1 g, Sigma Aldrich, Darmstadt, Germany). Eye drops of 1% atropine sulfate (Allergan, Westport, IR) and 4 mg/mL of oxybuprocaine chlorhydrate (Novesin. Laboratoires Thea Pharma spa, Milan, Italy) were applied for mydriasis and local anesthesia, respectively.

The body temperature was maintained at 37 °C with a heat pad. Flashes were provided by the Ganzfeld dome (Biomedica Mangoni, Pisa, Italy), and electrical signals were simultaneously acquired from both eyes with a gold electrode loop placed on each cornea. Electrical references were subcutaneously inserted into the anterior scalp. Traces of 300 ms were induced by flashes with increasing light intensities (0.001–100 cd×s/m^2^ range) and amplified differentially (bandpass filtered at 0.3 to 300 Hz). Lastly, digitized signals were analyzed by measuring the a-wave and b-wave with procedures based on Igor Pro 8 software (Wavemetrics Inc. 10200 SW Nimbus, G-7 Portland, OR 97223, USA), compiled by Dr. Stefano Di Marco. Statistical tests and plots were performed by Prism7 software (2365 Northside Dr. Suite 560San Diego, CA 92108, USA).

### 2.4. Western Blotting

Retinas of C57BL/6J mice subjected to ERG recordings, used as samples for this analysis, were extracted and lysed with the modified RIPA buffer, described by Piano et al. (2013) and the proteins quantified with the Bradford assay. Total protein (25 μg) was loaded for each sample in the pre-cast 4–20% polyacrylamide gel (Mini-PROTEANR TGX gel, Bio-Rad) and transferred to PVDF membranes (Trans-BlotR TurboTM PVDF Transfer packs, Bio-Rad). The blocking phase was carried out with 5% milk (Blotting Grade Blocker Non-Fat Dry Milk, Bio-Rad) prepared with T-TBS 1x (20 mM Tris-HCl, PH 7.5, 150 mM NaCl and 0.1% Tween 20) for 1 h, to prevent the formation of non-specific binding with antibodies. The membrane was then incubated with the primary antibody (Table 1) diluted in 5% milk, overnight at 4 °C. After 3 × 10 min washes in T-TBS, the membrane was incubated with the secondary antibody, conjugated with peroxidase (HRP) (Table 1) diluted in 5% milk for 2 h at room temperature.

Bands were visualized using an enhanced chemiluminescence substrate detection system (LuminataTM Forte Western HRP Substrate, Millipore Darmstadt, Germany). Luminescent images were acquired via LAS 4010 (GE Healthcare Life-Sciences, Pittsburgh, PA, USA). Densitometry was performed off-line using ImageJ software.

Each protein analyzed for this study was normalized to the reference protein values (GAPDH), using the stripping procedure, performed with 4 × 10 min washes in glycine buffer (pH 2), followed by 2 × 10 min washes in T-TBS and blocking in 5% milk for 1 h. The incubation of primary and secondary antibodies (Table 1) was performed with the same protocol described above.

### 2.5. Immunohistochemistry

Explanted eyes were fixed in 4% paraformaldehyde. then embedded in OCT with the correct orientation (Tissue-Tek O.C.T. Compound, Sakura Finetek Italia, srl, Mestre, Italy) and stored at −20 °C. Frozen retinal sections were obtained with a cryostat (Leica Biosystem srl, CM1850 Milan, Italy). Selected cryo-sections from the central retina were washed for 3 × 10 min in PBS, then incubated for 45 min in blocking solution (BSA 1%, Triton-X100 0.3% in PBS) at room temperature. Subsequently, the sections were incubated with primary antibodies (Table 2) overnight at 4 °C diluted in 1% BSA solution and 0.03% Triton-X100 in PBS. Sections were then washed for 3 × 10 min in PBS and incubated with related secondary antibodies (Table 2) for 2 h at room temperature. Finally, nuclei were stained with basic dye (DAPI, Merck) and then sections were covered with a mounting medium (Vectashild^®^ Vector Laboratories, Burlingame, CA, USA).

The images were obtained with a Zeiss fluorescence microscope equipped with ApoTome technology (Zeiss Microscopy, Jena, Germany) and analyzed with ImageJ software.

### 2.6. TUNEL Technique

Dying cells were labeled using the standard protocol of the terminal deoxynucleotidyl transferase dUTP nick end labeling (TUNEL technique) [37]. Tunel+ cells in the outer nuclear layer (ONL) were manually counted using an optic microscope. Means of counts were analyzed and plotted with Prism7 software.

### 2.7. Statistical Analysis

Statistical comparisons for ERG registrations and immunohistochemistry evaluation of Saffron experiments were carried out with one-way or two-way variance of analysis (ANOVA test), followed by *t*-tests with Bonferroni or Dunnett correlation; Statistical comparisons for ERG registrations and WB results of Naringenn experiments were carried out with the Wiloxon-Mann-Whitney non parametric test, using the Origin Lab 8.0 (MicroCal, Northampton, MA, USA) and GraphPad Prism 9.

## 3. Results

To validate the idea of a possible correlation between treatment and specific pathological profile, in this work we tested different nutraceutical compounds in different models of genetic origin, where oxidative stress plays a secondary role in the progression of the pathology, and in ageing where oxidative stress plays a primary role.

### 3.1. Royal College of Surgeons Rat (RCS)

**Figure 1:**
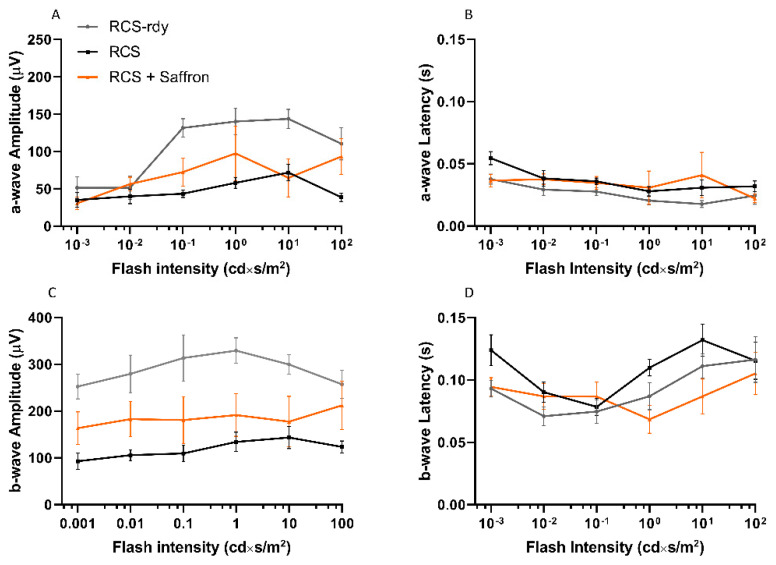
Impact of the Royal College of Surgeons Rat (RCS)Saffron treatment on the flash electroretinogram response. (**A**–**D**) Light dependent responses from P30 rats (*n* = 9). No treatment-related significant differences were found in a- or b-wave amplitudes or in their related latencies. Neuroprotective properties of saffron seem to have been overcome by an extreme severity of this retinal dystrophy. (±SEM; Statistical tests two-way ANOVA followed by Dunnett’s test).

We tested the effect of saffron treatment in an animal model of inherited retinal dystrophy, RCS rats, commonly used as an in vivo representative model of RP [38]. Recently, saffron was tested in both an animal model and in patients where oxidative stress plays an important role in the initiation of the degenerative process; with clear and important results [31]. The RCS rat has an inherited and spontaneous retinal dystrophy caused by the mutation of the Mertk gene. This mutation causes a dysfunctional receptor tyrosine kinase that leads to impairments in the disc shedding of photoreceptors (both cones and rods). The outer segments of photoreceptors build up in the sub-retinal space inducing a severe degeneration of photoreceptors, leading to complete blindness at P60 [39]. In RCS rats, there is a progressive depletion in the electrical retinal response due to the rapid death of photoreceptors [40]. In the current study, to assess the effects of saffron treatment, the retinal function was monitored by flash-ERG at early developmental stages (P20, P24, P27, P30, P35). Retinal electrical traces were analyzed by measuring “a” and “b”-wave amplitudes and their related latencies. Figure 1 shows results for a- and b-waves as a function of increasing luminance at P30. As control rats, we used congenic (RCS-rdy). The differences between treated and untreated rats were not significant.

**Figure 2:**
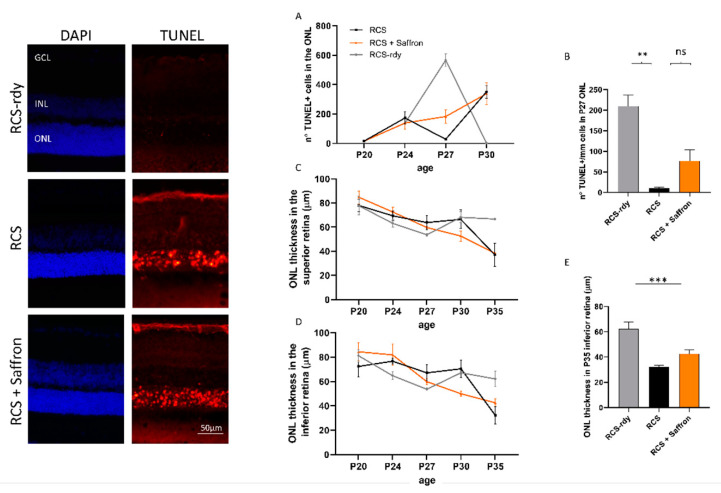
Impact of the Royal College of Surgeons Rat (RCS) Saffron treatment on TUNEL-positive cells in the outer nuclear layer (ONL) during retinal development. Representative pictures of nuclei-stained retinal layers (DAPI) and dying photoreceptors (TUNEL technique) at P30. (**A**) TUNEL+ cell count at different developmental stages (**B**) TUNEL+ cell count at P27. (**C**,**D**) Means of ONL thickness at different developmental stages in superior and inferior retina, respectively. (**E**) Bar graph of ONL thickness at P35 in inferior retina. (*n* = 4; ±SEM; Statistical tests: two-way ANOVA followed by Dunnett’s test) significance indicator: n.s. *p* > 0.05; ** *p* < 0.01; *** *p* < 0.001.

Saffron treatment (1 mg/kg/day) does not appear to help in maintaining retinal function. Morphological analysis at the same age is reported in Figure 2. Transversal sections of stained retinal nuclei (DAPI, in blue) or labeled dying neurons (TUNEL technique, in red) are shown at P30. At this stage, the physiological cell death in the retina is completed in RCS-rdy rats, while in RCS rats there is a continuous retinal cell death with a flex at P27 [41]. Graph A in Figure 2 summarizes the cell death rate in the ONL over time. The saffron treatment (orange line) can barely modulate cell death at P27 (Figure 2B) and results in a physiological trend similar to the RCS-rdy rats (blue line). In parallel to the rate of the photoreceptors’ death, graphs C and D in Figure 2 show the relative ONL thinning over time. While in the RCS-rdy group (blue line), the ONL thickness remains stable after the developmental phases, in the RCS rats there is a progressive retinal thinning which primarily affects the superior retina [42]. Therefore, saffron treatment slightly mitigates the dystrophy in the less affected retina (the inferior retina) at least up to P35 (Figure 2E).

**Figure 3:**
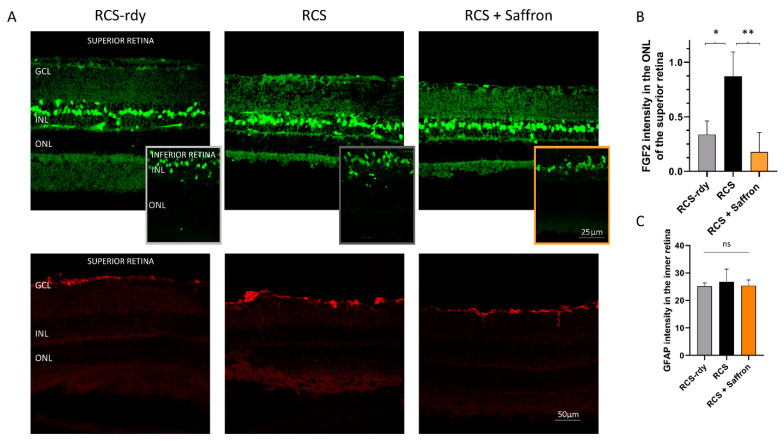
Impact of the of RCS Saffron treatment on the FGF2 and the GFAP expression. (**A**) Representative retinal images at P30, immuno-labelled against FGF2 (green) and GFAP (red). (**B**) FGF2 positive pixels in the ONL of treated, untreated, and control retinas; (**C**) GFAP analysis of treated untreated and control retinas. GCL: ganglion cell layer; INL: inner nuclear layer; ONL: outer nuclear layer (*n* = 4; ±SEM; Statistical tests: One way ANOVA followed by Dunnett’s test) significance indicator: n.s. *p* > 0.05; *p < 0.05; ** *p* < 0.01.

It is well known that Müller cells have an essential role in supporting and resolving conditions of retinal stress. In particular, they are highly sensitive to retinal injury and immediately express increased levels of glial fibrillary acidic protein (GFAP). Therefore, this molecule has been considered an early marker of retinal stress [8,43]. Müller cells release trophic factors, such as the Fibroblast growth factor 2 (FGF2) to photoreceptors, as well as proinflammatory cytokines. In RCS rats, Müller cells are involved in compensatory pathways, which trigger an increase in their number, hypertrophy, and GFAP expression around P30 [44]. Figure 3A shows representative images of transversal superior retinal sections immuno-labeled for FGF2 (green) and GFAP (red) expressed by Müller cells. The saffron treatment significantly reduces the FGF2 released in the ONL in the superior retina (see Figure 3B). The GFAP analysis does not present similar differences (see Figure 3C).

Results showed that the saffron treatment is unable to cope with degenerative processes in RCS efficiently.

### 3.2. Fischer-344 Rats (Fischer)

**Figure 4:**
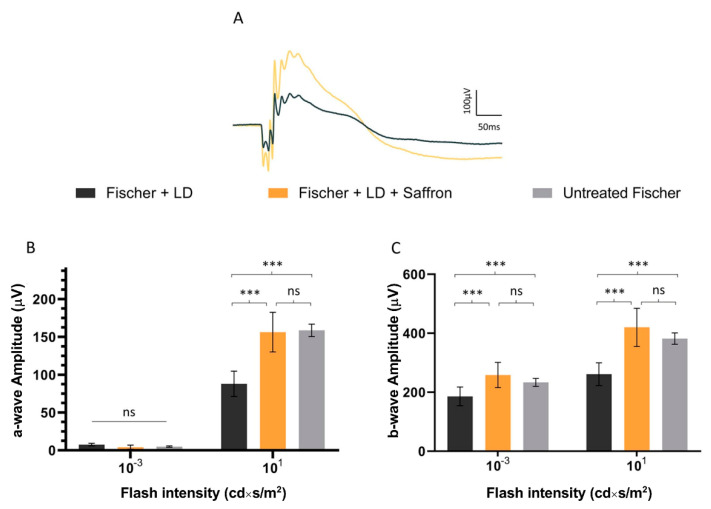
Saffron treatment on the flash electroretinogram response in treated and untreated light damage (LD) Fischer rats. (**A**) Representative traces of the scotopic electroretinographic response in Fischer rats. (**B**) A-wave amplitude at 0.001 cd×s/m^2^ and 10 cd×s/m^2^; (**C**) b-wave amplitude at 0.001 cd×s/m^2^ and 10 cd*s/m^2^. n.s. p>0.05; *** *p* < 0.001; (dark gray: Fischer + LD; orange: Fischer + LD + Saffron; light gray: Untreated Fischer; *n* = 6; ±SEM; Statistical tests Two way ANOVA followed by Dunnett’s test).

Fischer-344 albino rats present spontaneous age-related dystrophy, which targets many tissues such as the cornea, renal tubules, and vascular internal laminae [45,46]. Furthermore, several studies describe retinal age-dependent impairments with a progressive reduction in visual evoked potential amplitude and electroretinographic responses [47] as well as a continuous loss of photoreceptors in the peripheral retina [48] probably due to Müller cell dysfunction [49]. On the other hand, Sprague Dawley (SD) albino rats do not present any spontaneous degenerative process and have mainly been used as a simplified AMD model where retinal degeneration is induced by light exposure. Photoreceptor death is caused by oxidative stress (photooxidation of membrane lipids). Fischer-344 rats are also albino, and supposedly retinal degeneration is induced by exposure to high-intensity light as in SD. We wonder whether saffron treatment after LD has the same efficacy in SD and Fischer-344 rat retina. Figure 4 shows the a- and b-wave responses in dark-adapted flash ERG recordings, in Fischer rats, following the same protocol developed in SD, of the LD and saffron treatment groups [50]. After 15 days of recovery, saffron can preserve both a- and b-wave in Fischer rats with particular efficiency for the a-wave as shown at high-intensity flashes (Figure 4 B:10 cd*s/m^2^).

**Figure 5:**
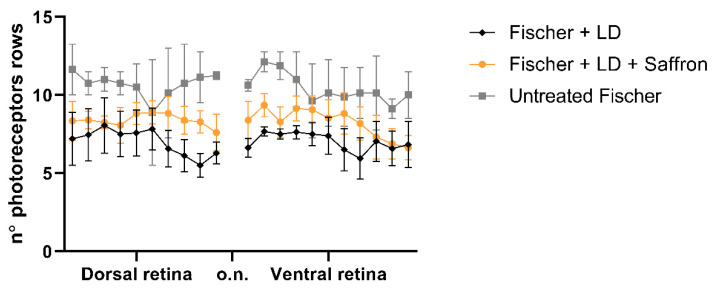
Photoreceptors rows. Count of photoreceptor rows in the superior and inferior retina in control and treated and untreated light damage (LD) Fischer rats (rats *n* = 6 each group; ±SEM; Statistical tests: TWO way ANOVA followed by Dunnett’s test. Fischer + LDvsFischer + LD + Saffron *p* < 0.001; Fischer + LDvsUntreatedFischer *p* < 0.001).

**Figure 6:**
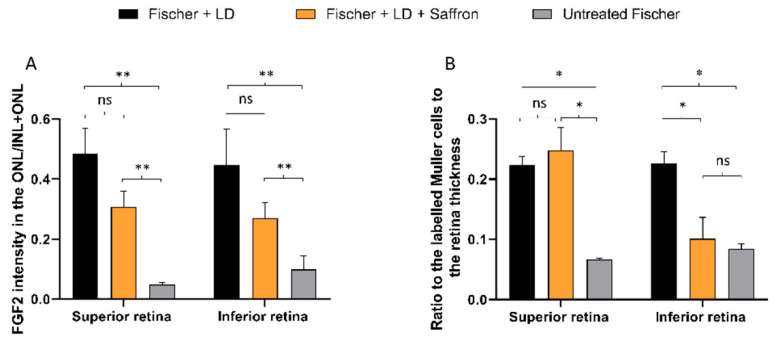
FGF2 and GFAP modulation by Saffron treatment of Fischer rats. (**A**) Quantification of FGF2 positive pixels in the ONL respective to the total amount in the retina layers. The graph bar shows no significant reduction of the FGF2 expression in treated rats (orange) compared to the LD control (black). (**B**) Quantification of GFAP positive Müller cell branches normalized to retinal thickness. The graph bar shows a significant reduction of the GFAP ratios in the inferior retina of Fischer treated rats (orange) compared to the relative LD control (black). (*n* = 6; ±SEM; Statistical tests: One way ANOVA followed by Dunnett’s test) significance indicator: n.s. *p* > 0.05; * *p* < 0.05; **p < 0.01.

**Figure 7:**
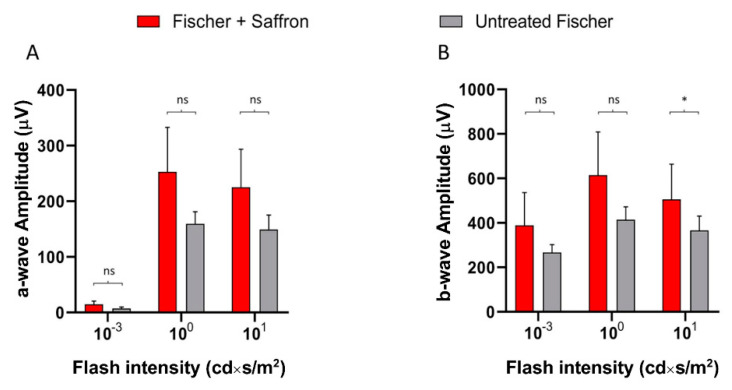
ERG response in saffron treated and untreated Fischer rats. (**A**) A-wave amplitude at three luminances (0.001 cd×s/m^2^, 1 cd×s/m^2^, 10 cd×s/m^2^) in treated (red) and untreated (grey) rats. (**B**) B-wave amplitude at three luminance in treated (red) and untreated (grey) rats. B-wave amplitude is statistically significant only at high intensity flash (10 cd*s/m2. (*n* = 3; mean ± SEM; Statistical tests: One way ANOVA followed by Dunnett’s test) significance indicator: * *p* < 0.05.

Interestingly Fischer rats are less responsive to light stimuli compared to SD rats (see for ref. [31]); in particular, the b-wave seems to be more compromised, probably in relation to Müller cells dysfunction [51]. In contrast, Fischer rats seem to be less sensitive to light damage [52]. In particular, the ONL thickness in these animals after light exposure is homogeneously reduced without the presence of the “hotspot”; the particularly sensitive area that generally appears in the superior SD retina [53] (Figure 5). An additional interesting observation is that the Fischer retina appears not to activate many stress responses. Figure 6 shows quantification of a stress marker, GFAP, expressed by Müller cells; light exposure induces an up-regulation of GFAP which is reduced by saffron treatment in the less sensitive area (the inferior retina); while FGF2 which is highly expressed by SD after LD in the ONL and modulated by saffron [36] is moderately increased by LD in Fischer rats and slightly modulated by saffron. Interestingly beneficial saffron effects are clearly detectable in the electrical response of retinas in animal treated without LD (Figure 7). It has to be noted that saffron treatment in healthy SD rats does not modify retinal responses (Supplementary Figure S1). It seems possible to conclude that saffron treatment can cope with the degenerative process itself and it is efficiently able to reduce the impact of an additional environmental stress.

### 3.3. Aging

In the physiological senescence processes, the mechanisms that trigger cell malfunction and, eventually, degeneration have a much slower progression than in diseases such as RP which have relevant genetic components, or in age-related multifactorial diseases. For this reason, early prevention with the intake of nutraceutical molecules could represent an effective strategy for preserving the viability of retinal cells. This idea is also based on the observation that with aging, oxidative stress and neuroinflammation progressively increased.

**Figure 8:**
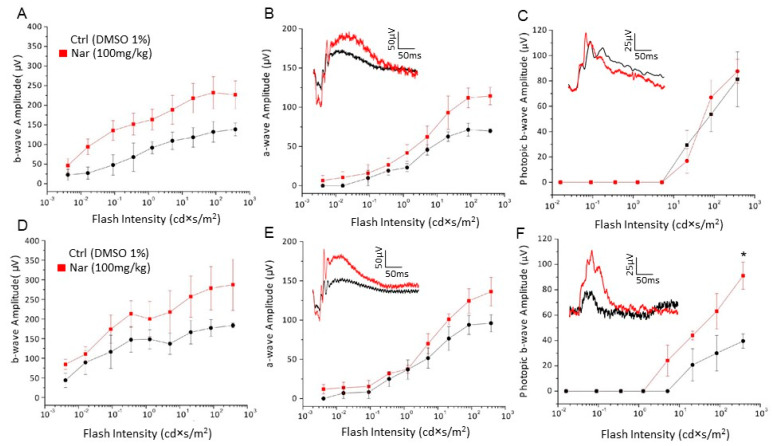
Naringenin preserves retinal function in older C57BL/6J mice. (**A**–**C**): Retinal function tested by recording scotopic and photopic ERG in 9-month-old mice treated with naringenin 100 mg/kg/day for three months. The curves show the amplitude respectively of scotopic b-wave (**A**) and a-wave (**B**) and photopic b-wave (**C**); (**D**–**F**): Retinal function tested by recording scotopic and photopic ERG in animals of 12 months treated with naringenin 100 mg/kg/day for six months. The curves show the amplitude respectively of scotopic b-wave (**D**) and a-wave (**E**) and photopic b-wave (**F**). The values shown represent the mean ± SEM; *n* = 6 for each group; Wilcoxon-Mann-Whitney Test statistical analysis (* *p* ≤ 0.05).

Functional, biochemical, and morphological analyses were performed at the end of the treatment with 100 mg/kg/day of naringenin in two groups of C57BL/6J animals 9 or 12 months old and compared with the respective control group treated with the vehicle alone (DMSO 1%). Figure 8 shows the scotopic and photopic ERG responses in animals of 9 (Figure 8A–C) and 12 months (Figure 8D–F) treated with naringenin and compared with aged-matched mice treated with the vehicle alone. The data carried out from animals at 9 months (Figure 8A–C) show that the chronic treatment with naringenin only partially preserves the visual function. It is possible to observe only a tendency to increase the amplitude of both the a- and b-wave of the scotopic ERG, while the photopic b-wave amplitude curves are substantially superimposable. On the contrary, in the case of the animals analyzed at 12 months of age (Figure 8D–F), a partial recovery of the scotopic ERG is still observed while for the ERG recorded in photopic conditions a significant increase in the amplitude of the b-wave is evident, indicating a higher functionality of the cones. Interestingly, the efficacy of the treatment seems to increase with time; the longer, the better.

**Figure 9:**
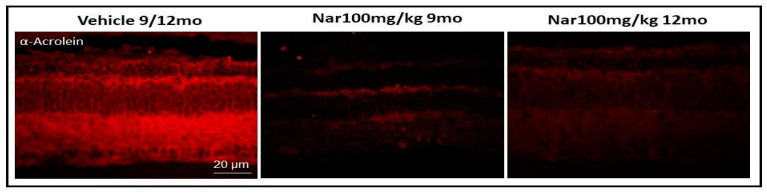
Naringenin reduces lipid peroxidation levels in older C57BL/6J mice. The figure shows the retinal section obtained from animal treated with vehicle alone or with naringenin 100/mg/kg/day respectively for three or six months. Red staining was obtained with the antibody anti-acrolein.

The results obtained for functional analysis may be due to the reduction of ROS concentration in the retinal cells following treatment with naringenin. Figure 9 shows sections of retinal tissue obtained from control animals and treated with naringenin. In the latter, it is possible to observe the marked reduction of the red staining obtained with anti-acrolein antibody, a specific marker of the formation of adducts of lipid peroxidation.

**Figure 10:**
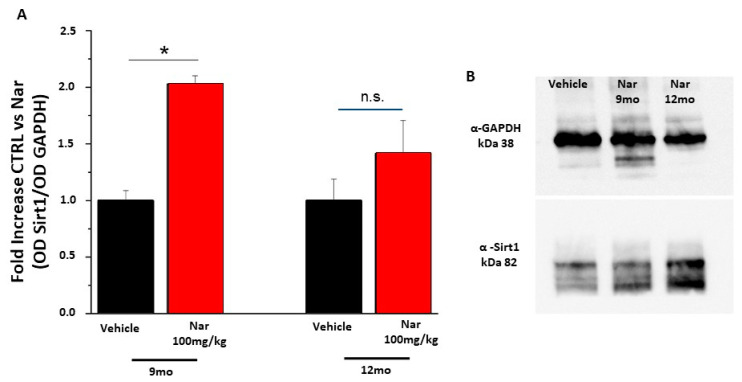
Naringenin preserves the levels of Sirt1 in older C57BL/6J mice. (**A**) The bar graph shows that naringenin treatment is able to preserve the levels of Sirt1. This increase is significant in the animals treated for three months (red bar) respective to the relative control (black bar); in the animal treated for six months (red bar) this increase is again present although it does not reach statistically significant values. (**B**) Representative example of a single western blotting experiment. The values shown represent the mean ± SEM; *n* = 3 for each group; Wilcoxon-Mann-Whitney Test statistical analysis (* *p* ≤ 0.05).

**Figure 11:**
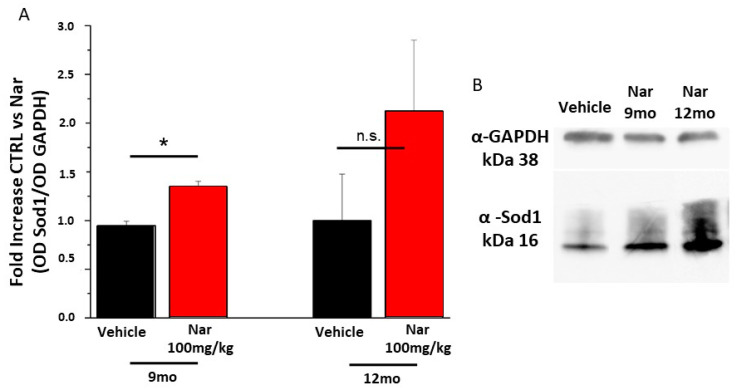
Naringenin preserves the levels of Sod1 in older C57BL/6J mice. (**A**) The bar graph bar shows that naringenin treatment is able to preserve levels of Sod1. This increase is significant in both groups of animals treated for three or six months (red bar), respective to the relative control (black bar). (**B**) Representative example of a single western blotting experiment. The values shown represent the mean ± SEM; *n* = 3 for each group; Wilcoxon-Mann-Whitney Test statistical analysis (* *p* ≤ 0.05).

Finally, to assess whether the action of naringenin was due to scavenger activity against ROS or to the activation of specific cellular pathways, a semi-quantitative western blotting analysis was performed for proteins involved in the detoxification process. Figure 10 shows the data obtained for Sirt1, a regulatory protein upstream of the Sod-mediated detoxification system. The graph bars in Figure 10A and the example in panel 10B show that the levels of Sirt1 increased significantly in the 9-month mice treated with naringenin compared to the control animals (*p < 0.05); the same trend is true also for the 12-month-old mice. This result is in accordance with the data obtained from the effector protein Sod1 shown in Figure 11A, B. The graph bars in Figure 11A shows a significant increase in Sod1 levels in 9-month-old mice and the same tendency in 12-month-old animals, following treatment with naringenin, indicating a recovery of the detoxifying activity of the cells which results in a reduction of ROS and improves functionality.

Together, these results demonstrate the efficacy of chronic treatment with naringenin as a nutraceutical molecule in preventing cellular senescence and redox imbalance processes, preserving the functionality of the photoreceptors and slowing down vision loss.

## 4. Discussion

The data reported in this work provide a new perspective on the use of nutraceutical molecules as a possible therapy and/or in the prevention of various retinal pathologies. What emerges is that treatments with different nutraceutical molecules, specifically naringenin and saffron, are differentially effective in distinct models of RP and age-related neurodegenerative retinal processes, indicating that the efficacy is related to the characteristics and the time course of the neurodegenerative progression. We assumed that, at least for naringenin, the main pathway on which this molecule intervenes is stress-oxidative; moreover, the efficacy of the treatment is also linked to the moment when oxidative stress starts. Saffron was initially tested in a light damage model where membrane lipid peroxidation [54] represents the starting event in the degenerative cascades. In this model and in patients (AMD and Stargardt) [28,29,30,31] where oxidative stress and neuroinflammation play pivotal roles, the saffron treatment appears efficient in slowing down and/or blocking neurodegenerative progression. Microarray experiments [55] have suggested a complex pattern of possible ways of action, some of which have been tested [24,56,57]. The same treatment gave a different outcome in an RP model, RCS rats, which has a mutation at the level of the RPE cells that causes the simultaneous death of rods and cones due to the lack of phagocytosis of photoreceptor outer segments. Oxidative stress plays a marginal role in the progression of the disease, as it does for neuro-inflammation. Saffron treatment was unable to maintain retinal function.

Nevertheless, it was possible to detect a small reduction in photoreceptor apoptosis and a control in FGF2 expression, particularly in inferior retina, confirming saffron’s multiple ways of action. In any case, a different therapeutic approach has to be used for this disease. On the other hand, in a neurodegenerative model induced by a mix of spontaneous dystrophy and light exposure, as in Fischer rats with LD, saffron confirms its capacity to maintain morphology and function in response to a degeneration acutely induced by oxidative stress. Furthermore, saffron is also useful in improving light response in “Untreated controls”, suggesting the possibility to cope with the supposed Müller cell dysfunction at an early stage. Therefore, an interesting perspective would be to clarify the effect of saffron on retinal glial cells to identify which pathways are most affected in mitigating neuro-inflammation. Other natural molecules (naringenin and quercetin) have been tested in an RP animal model where the mutation is at the level of the specific PDE6 gene of the rods, a pivotal enzyme involved in the phototransduction of these cells, which in any case are inexorably destined for death [12,13]. Treatment with naringenin and quercetin was effective in prolonging the survival of the cones, which generally die as a consequence of rod disappearance [17]. Despite some differences in the biological effect with respect to the RP model, treatment with naringenin has successfully prevented the cellular mechanisms of senescence in old wild type mice, where the visual function undergoes an age-dependent impairment of ERG responses when compared with young mice of the same strain (data shown in [17], Supplementary Materials). The results published by Piano et al. 2019, show how the treatment with naringenin brings the levels of the detoxifying enzymes Sod1 and Sod2 in rd10 animals towards the values found in healthy animals of the same age. This result indicates that in this RP model, naringenin has a prevalent scavenger action against ROS. Accordingly, the results obtained in aged C57BL/6J mice show an increase in the levels of the enzymes involved in antioxidant detoxification, Sirt1 and Sod1, following treatment with naringenin. It is known in the literature [58] that with increasing age, the protein levels of Sirt1 decrease, leading to a progressive inefficiency of cellular defenses against ROS. The results shown here regarding the effects obtained on the retinal tissue agree with data already published on the efficacy of treatment with naringenin in other tissues [58] and indicate that a long-term preventive and chronic protocol is effective at slowing down those cellular aging processes by inducing the physiological defenses of the cells. Further studies are needed to verify whether the ways of actions of naringenin include the ability to control gene expression as was proven for saffron treatment [55].

## 5. Conclusions

In conclusion, treatment with natural molecules appears quite promising, although relevant issues have been raised: (1) It is impossible to generalize. The efficacy of each treatment is related to the pathology, its origin, and its progression and this aspect has to be thoroughly evaluated; (2) The comparison between different treatments must be performed using the same experimental model and protocol in order to find equalities and differences in the target pathways of the treatment and also to develop combined approaches; (3) There is a need to develop systematic screening that can predict the action and therapeutic potential of different compounds.

An additional comment comes from noticing [11] that oxidative stress plays a pivotal role in a variety of pathological processes in the entire nervous system, opening the possibility to use natural treatments to cope with a variety of neurological diseases. However, this has to be done only once in-depth knowledge has been acquired.

## Figures and Tables

**Table 1:** List of antibodies for Western blotting.

Antibody	Company	Work Dilution	Application
Sod 1	Sigma Aldrich	1:500	WB
Sirt 1	Merk Millipore	1:1000	WB
GAPDH	Sigma Aldrich	1:5000	WB
Anti-rabbit IgG HRP conjugated	Cell Signaling Technology	1:5000	WB
Anti-mouse IgG HRP conjugated	Merk Millipore	1:5000	WB

GAPDH: glyceraldehyde-3-phosphate dehydrogenas; WB: western blotting.

**Table 2:** List of antibodies for Immunohistochemistry.

Antibody	Company	Work Dilution	Application
Anti-acrolein	AbCam	1:1000	IH
Fibroblast grow factor 2 (FGF2)	Millipore	1:200	IH
Glial fibrillary acidic protein (GFAP)	Dako	1:1000	IH
Anti-rabbit Alexa Fluor^®^ 568	Sigma Aldrich	1:500	IH
Anti-mouse Alexa Fluor^®^	Molecular probes, Invitrogen Carlsbad	1:200	IH
Anti-rabbit Alexa Fluor^®^	Molecular probes, Invitrogen Carlsbad	1:200	IH

IH: Immunohystochemistry.

## Data Availability

The experimental data that support the figures within this paper and other findings of this study are hosted at the Istituto Italiano di Tecnologia and Department of Pharmacy, University of Pisa and can be accessed by contacting the corresponding author.

## Supplementary Materials

The following are available online at https://www.mdpi.com/2072-6643/13/3/770/s1, Figure S1: Impact of Saffron treatment on the flash electroretinogram response of SD (Sprague Dawley) rats.
