# Supplementary material for: Retinal Neurodegeneration: Correlation between Nutraceutical Treatment and Animal Model
Source: Nutrients. 2021 Feb 27;13(3):770. doi: 10.3390/nu13030770 (PMC7997156; doi:10.3390/nu13030770)
Supplement: Supplementary file 1 [file nutrients-13-00770-s001.pdf]

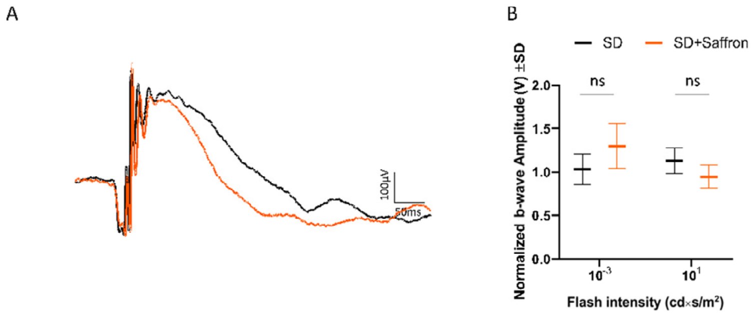

Figure S1: Impact of Saffron treatment on the flash electroretinogram response of SD (Sprague Dawley) rats. A) Representative ERG traces of untreated (black line) and treated (orange line) Sprague Dawley rats. B) b-wave amplitude at two luminance in treated (orange) and untreated (black) rats. No significant differences were found between groups. (*n*=3; ±SD; Statistical tests: ONE way ANOVA followed by Dunnett's test;ns=not significant 10^{-3}SDvs10^{-3}SD+Saffron *p*=0.1533; 10^{1}SDvs10^{1}SD+Saffron p=0.3812).
